# Statistical Comparison of Spatial Point Patterns in Biological Imaging

**DOI:** 10.1371/journal.pone.0087759

**Published:** 2014-02-05

**Authors:** Jasmine Burguet, Philippe Andrey

**Affiliations:** 1 INRA, UMR1318, Institut Jean-Pierre Bourgin, Versailles, France; 2 AgroParisTech, Institut Jean-Pierre Bourgin, Versailles, France; 3 INRA, UR1197, Neurobiologie de l’Olfaction et Modélisation en Imagerie, Jouy-en-Josas, France; 4 IFR 144, NeuroSud Paris, Gif-Sur-Yvette, France; 5 Université Pierre et Marie Curie (UPMC), Paris 06, Paris, France; Banner Alzheimer’s Institute, United States of America

## Abstract

In biological systems, functions and spatial organizations are closely related. Spatial data in biology frequently consist of, or can be assimilated to, sets of points. An important goal in the quantitative analysis of such data is the evaluation and localization of differences in spatial distributions between groups. Because of experimental replications, achieving this goal requires comparing collections of point sets, a noticeably challenging issue for which no method has been proposed to date. We introduce a strategy to address this problem, based on the comparison of point intensities throughout space. Our method is based on a statistical test that determines whether local point intensities, estimated using replicated data, are significantly different or not. Repeating this test at different positions provides an intensity comparison map and reveals domains showing significant intensity differences. Simulated data were used to characterize and validate this approach. The method was then applied to two different neuroanatomical systems to evaluate its ability to reveal spatial differences in biological data sets. Applied to two distinct neuronal populations within the rat spinal cord, the method generated an objective representation of the spatial segregation established previously on a subjective visual basis. The method was also applied to analyze the spatial distribution of locus coeruleus neurons in control and mutant mice. The results objectively consolidated previous conclusions obtained from visual comparisons. Remarkably, they also provided new insights into the maturation of the locus coeruleus in mutant and control animals. Overall, the method introduced here is a new contribution to the quantitative analysis of biological organizations that provides meaningful spatial representations which are easy to understand and to interpret. Finally, because our approach is generic and punctual structures are widespread at the cellular and histological scales, it is potentially useful for a large spectrum of applications for the analysis of biological systems.

## Introduction

It is a remarkable fact in biology that functions are closely linked to specific spatial architectures in both the plant and animal kingdoms, and at different spatial scales. Many molecular, histological and imaging techniques can be used and combined to reveal how biological structures are organized in three dimensions (3D) for a large range of resolutions [1]. However, the lack of objective and quantitative methods devoted to the analysis of the images produced is a recurrent problem reported in the scientific community, and these data are still widely qualitatively analyzed through visual inspection [2], [3]. This is particularly the case when addressing the specific question of spatial organizations. At the cellular scale, for example, Duong et al. [4] noted that few statistical approaches are designed to assess intracellular organizations. At the histological scale, Da Silva-Buttkus et al. [5] emphasized that biological patterns are widely compared on the basis of visual similarity/dissimilarity, and regretted the lack of statistical tests to determine whether observed differences are significant or not. In general, the development of reliable methods for the statistical analysis of spatial organizations in biological images remains an important ongoing challenge.

In this context, we more specifically addressed the question of the statistical comparison of two spatial organizations in 3D. In this paper, we focus on data that can be assimilated to sets of points in a finite volume, e.g., positions of endosomes in a cell or of cells in a neuronal population. Inferring the way in which points in a punctual pattern are distributed in 3D is not a simple issue. The most commonly used approaches at this time involve the construction of point intensity maps (i.e., of the number of points per unit volume). This is equivalent to assuming that each point pattern is generated by an unknown point process (i.e., a stochastic process that produces sets of points), which is assessed by evaluating intensity variations in space. Based on 3D histograms of cell counts [6]–[9] or kernel density estimates [10], most of the proposed methods were designed to process single point patterns only.

To take inter-individual and experimental variability into account, biological data are replicated, resulting in a set of point patterns, which constitutes a sample from the same unknown point process. Although the consideration of replicated data is required to perform further statistical analysis, few studies in the literature deal with repetitions to assess a point distribution. In our opinion, one major obstacle that may explain the scarcity of proposed methods is the need for a spatial normalization procedure capable of placing repeated data in a common spatial framework. Actually, this step consists in removing the part of variability attached to the spatial domain containing the data (e.g., the size and shape of brains or of cells in different individuals). In a recent paper that addressed the spatial organization of endosomes in mammalian cells [11], this problem was circumvented by limiting itself to a pre-determined shape for cells, using micro-patterning techniques [12], [13]. Point patterns were then superimposed so as to be reduced to the simple case of a single pattern. Note that, as a consequence, individual specificity is lost and a bias may be introduced due, for example, to the dominance of patterns composed of a high number of points. Generally speaking, it is not possible to experimentally impose a standardized shape for cells, tissues or organs to be analyzed. We therefore took advantage of a spatial normalization method adapted to biological structures [14], [15], freely available in Free-D software [16]. All biological data considered in this paper were normalized beforehand using this procedure so that point coordinates in all sample patterns were expressed within the same 3D coordinate frame.

Within this standardized coordinate system, the number of points and their positions vary across patterns. The punctual nature of the data, with no point-to-point correspondence, is an obstacle to the integration of data based on averaging, as can be done for example in human functional neuroimaging studies (see [17] and [18] for a review of related techniques). To compute statistical maps of point intensity (i.e., number of points per unit volume) from a point pattern sample, we proposed in [19] an approach based on the theory of spatial point processes [20], [21], a statistical framework for the analysis of point patterns [22]–[28]. Our solution was based on a local point intensity estimator computed from the distances to the 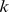
th nearest neighbors. By repeating estimations over a spatial grid, we were able to build a statistical map of intensity variations. By superimposing iso-intensity surfaces extracted from different maps, it was possible to visually compare spatial organizations in 3D. This strategy was successfully applied to unmask differences in the spatial organization of neuronal populations [29], [30].

Although informative, such visual comparisons have remained qualitative. Few strategies have been proposed in the literature to assess whether or not two point processes defined over a finite volume differ. Available statistical methods determine whether two point pattern samples may have been generated by the same point process or not [4], [31]. Although this could provide deep insights into the biological functions that are involved, to our knowledge, no approach exists that would allow us to reveal local domains that show significant differences between two point processes based on repeated data. Kelsall and Diggle [32] proposed a non-parametric approach to map the relative risk of two diseases based on 2D point intensity ratios, but this approach was limited to the comparison of single patterns. We introduce here a method to compare point processes based on repeated point patterns. We take advantage of the parametric framework established in [19] for intensity estimation and we introduce a test to compare local point intensities. By systematically repeating this statistical test in space, comparison maps are computed in order to reveal spatial domains where local intensities differ. Simulated data were used to analyze and characterize the method. Its application to the comparison of neuronal populations demonstrated its ability to objectively localize differences between punctual biological structures.

## Methods

A stochastic mechanism whose realizations are point patterns in 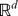 is referred to as a spatial point process. We develop our strategy in the case where 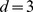, but it can be generalized to other dimensions. We assume that we are given two samples of point patterns, and we aim at comparing the spatial organizations of the two point processes that generated them. To do this, we propose comparing local point intensities.

Our approach is based on the point intensity estimator derived in [19] as follows. In the neighborhood of any position 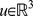, we approximate the local point distribution by a homogeneous Poisson process, a reference model that corresponds to the complete spatial randomness (CSR). The sole parameter of this model is the point intensity 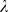. Under the local CSR assumption, the probability density function of the distance 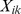 between 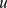 and its 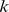
th nearest point in the 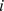
th point set of the sample is:
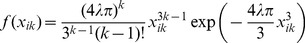
(1)


Based on the maximum likelihood method, the following unbiased estimator of 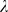 at position 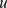 was obtained:
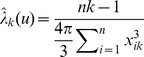
(2)where 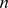 is the sample size [19]. Note that the only parameter of this estimator is the neighbor rank 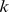. Then, given a spatial position 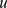 and two samples generated by unknown point processes, we propose a statistical test to compare point intensities at 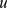. Under the CSR hypothesis, considering Equation 1, the probability density function of 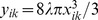 is



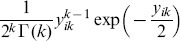
where 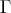 denotes the gamma function. Then 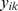 is distributed according to a 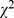 distribution with 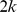 degrees of freedom. Considering the expression of 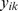 and Equation 2, the quantity 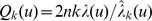 follows a 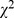 distribution with 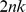 degrees of freedom.

Let us consider two point processes, and 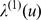 and 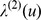 denote point intensities at 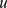 in the first and second processes, respectively. Let 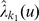 and 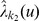 be the estimates of 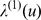 and 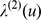, respectively. By definition, the ratio:
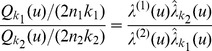
(3)follows a Fisher-Snedecor (FS) distribution with parameters 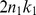 and 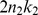, where 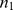 and 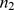 are the sizes of the first and second samples, respectively (see [33] for an introduction to the FS distribution). Testing the null hypothesis, 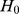: 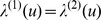, is then reduced to the analysis of:
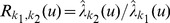
(4)relative to the distribution 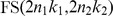. Therefore, under the local CSR assumption, given a fixed risk 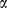, corresponding limit values 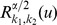 and 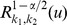 define the following intervals for 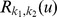:



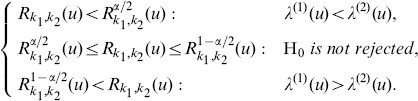



**Figure 1:**
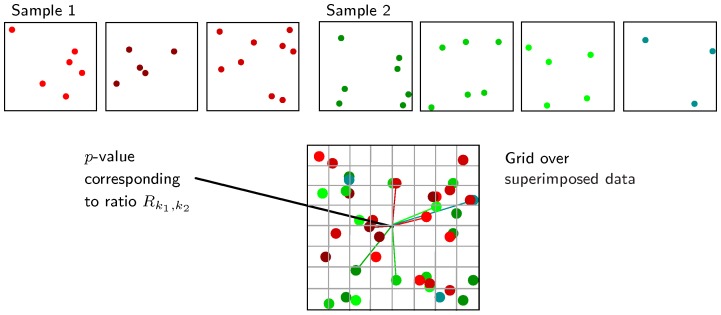
Comparison mapping method. Top: two samples of sizes 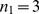 (left, red sample) and 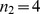 (right, green sample). Bottom: comparison map built from the samples. Values of ratio 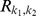 (see Formula 4) and corresponding 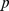-values are evaluated at each node of a grid superimposed over the data, based on distances to the 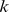-th nearest neighbors in Sample 1 (red segments) and in Sample 2 (green segments) (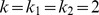).

The ratio 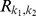 is evaluated at each node of a 3D grid covering the domain of interest, and 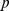-values corresponding to the distribution 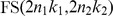 are determined (see Fig. 1). The resulting spatial map of 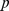-values is referred to as a 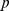-map. By converting 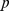-values into gray values, it is possible to visualize the spatial regions that exhibit the most significant differences. Isosurfaces obtained by thresholding the 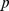-map at 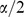 and 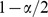 are an effective way to visualize domains in 3D where intensities statistically differ at the risk 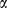.

## Results

### Application to Simulated Data

The comparison method was evaluated using synthetic data for which the exact intensity at any given position was known. First, point patterns were simulated according to homogeneous Poisson processes with various intensities, so that the CSR assumption was satisfied. To evaluate the robustness of the method to departure from the CSR condition, samples were also simulated using heterogeneous Poisson processes, so that the intensity varied according to the position. For this, 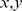 and 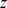 point coordinates were drawn from Gaussian distributions with standard deviations 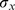, 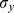 and 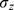, respectively.

In the following, intensities were compared using equal neighbor ranks, 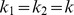, with 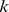 ranging from 2 to 16. In each case, first and second samples contained 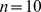 patterns each, with an average of 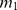 and 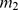 points per pattern, respectively. In the homogeneous case, points were generated over the centered unit cube, with 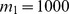 and 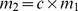 points, and 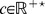. In the heterogeneous case, we fixed 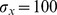, 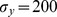 and 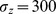 units, and we generated an average of 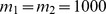 points per pattern, and centers of Gaussian distributions were possibly shifted.

#### Comparing local intensities at selected positions

To validate the local intensity comparison, the empirical distribution functions of 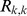 values were computed over 1000 repetitions, at selected positions 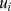, and compared to the expected distribution functions of 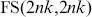 under the CSR assumption.

**Figure 2:**
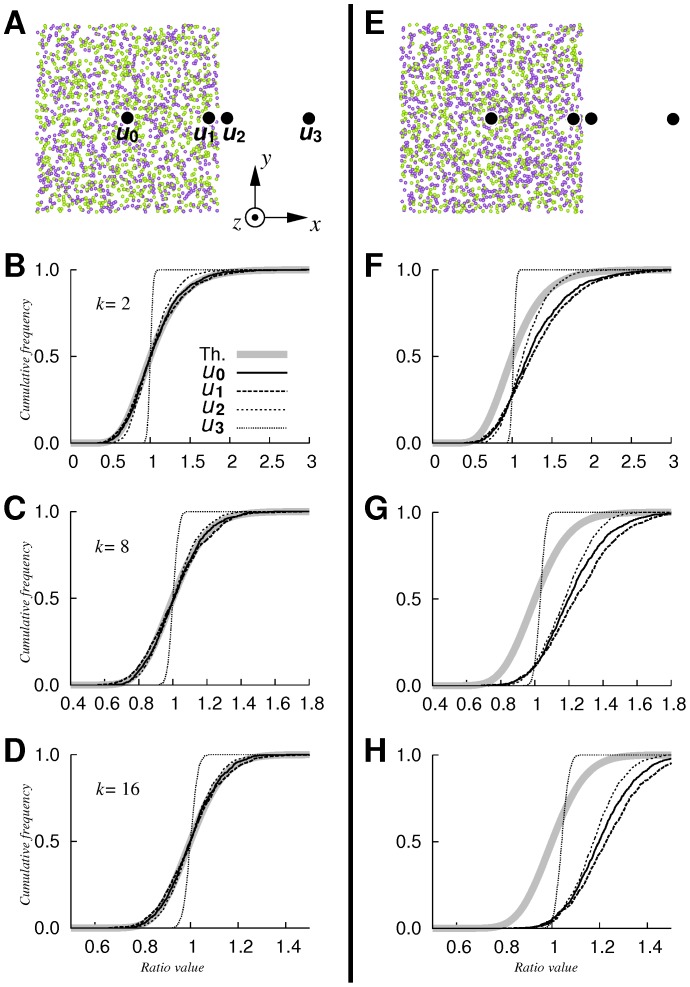
Local intensity comparison: homogeneous Poisson processes. Cumulative empirical functions of ratio 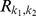 computed from pairs of simulated point pattern samples (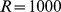 repetitions). A: typical patterns of Sample 1 (green) and Sample 2 (purple) simulated over the centered unit cube. Sample sizes 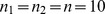, true intensity 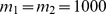 points/unit volume. Black dots: positions at 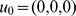, 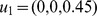, 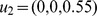 and 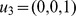. B–D: corresponding empirical functions computed at positions 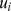, 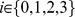, with 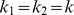 and 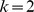 (B), 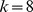 (C) and 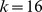 (D). Thick gray line: cumulative distribution function of 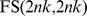. E–H: same as A–D, but with 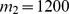 points/unit volume.

Results obtained for homogeneous point processes are shown in Fig. 2. For 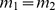 (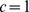), we observed the same global scheme, regardless of the value of 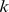 considered (Figs. 2A–D). First, at the central position 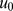, empirical and theoretical distribution functions coincided. This result was expected since the CSR assumption was true and 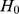 was satisfied. Concerning position 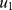, expected theoretical distances to the 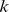-th nearest neighbor (0.074, 0.122 and 0.155 units for 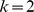, 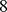 and 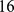, respectively) exceeded the minimal distance from 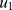 to the cubic domain border (0.05 units). Thus, regardless of the 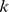 value, the local CSR assumption was no longer valid. Interestingly, empirical and theoretical functions at 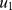 remained very close (Kolmogorov-Smirnov test, 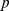-values of 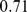, 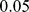, and 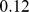 for 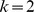, 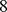 and 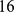, respectively). As for positions 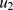 and 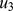, where the true intensity is null, 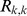 values tended to concentrate toward the medial value of the distribution function. Thus, the variability of 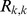 diminished, whereas the probability of not rejecting the null hypothesis increased. This was even more pronounced as the position moved further away from the points. This can be explained by the fact that in regions devoid of data, the estimator 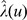 has a small variance and mainly reflects a systematic positive bias [19]. When intensities 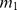 and 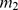 differed (see Figs. 2E, 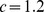), empirical functions at 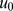 and 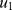 were translated toward greater values (see Figs. 2F–H), so that the probability to reject 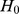 to the benefit of 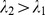 increased. Since 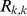 represents the ratio between intensity estimates, the magnitude of the gap for all 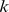 values tested logically reflected the ratio of intensities: it resulted in a shift of empirical values of approximately 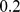 units toward greater values, which is consistent with the value of 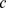. More precisely, on the basis of Equation 3, empirical ratios at 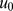 divided by 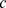 are distributed according to 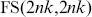. Concerning 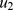 and 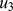, the variability of 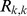 decreased as the evaluation position moved away from the point domain, which was similar to the case 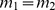 and for the same reason.

**Figure 3:**
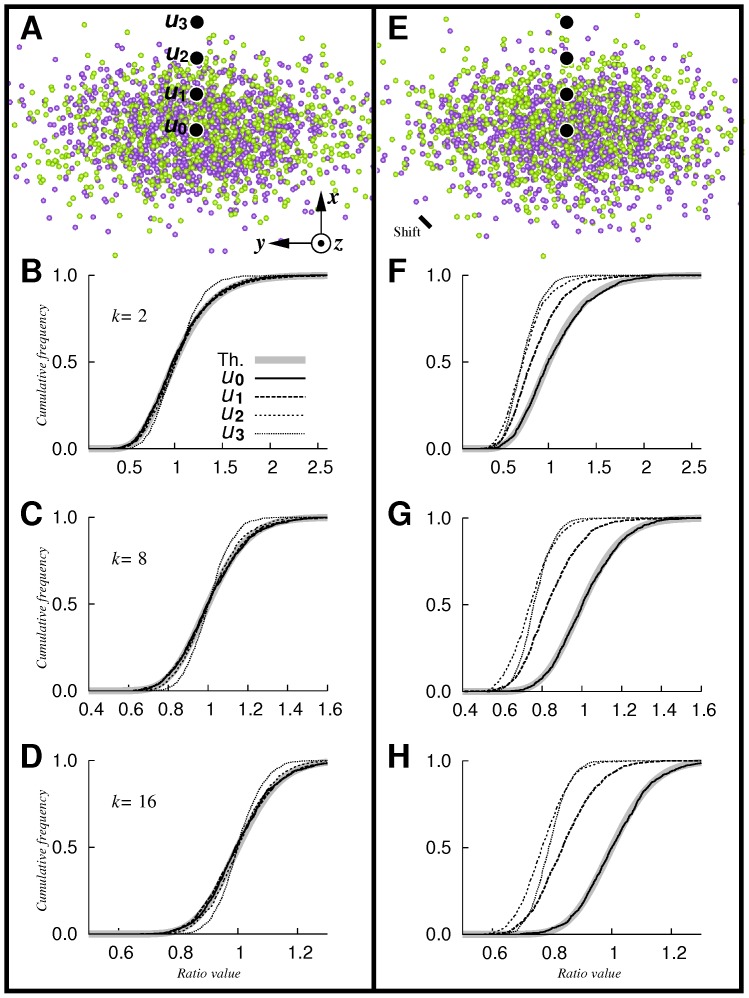
Local intensity comparison: heterogeneous Poisson processes. Simulated empirical functions of ratio 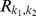 computed from pairs of simulated point pattern samples (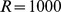 repetitions). A: typical patterns of Sample 1 (green) and Sample 2 (purple) drawn from identical centered Gaussian distributions of standard deviations 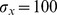, 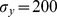 and 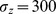 units. Sample sizes 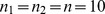, 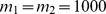 points per pattern in average. Black dots: positions at 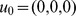, 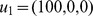, 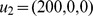 and 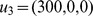. B–D: corresponding empirical functions computed at positions 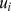, 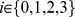, with 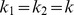 and 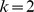 (B), 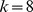 (C) and 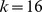 (D). Thick gray line: cumulative distribution function of 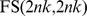. E–H: same as A–D, but centers of Gaussian processes generating Sample 1 and Sample 2 are 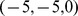 and 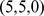, respectively.

The same analyses were performed for heterogeneous processes. Results are shown in Fig. 3. Interestingly, when 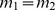 and with no shift between the two distributions (Fig. 3A), results were similar to the ones obtained in the case of identical homogeneous processes (see Figs. 3B–D). Within high intensity regions, theoretical and empirical distribution functions coincided for all of the 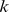 values tested (Kolmogorov-Smirnov test, 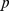-values of 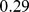, 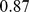, and 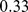 at 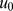, and 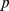-values of 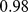, 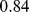, and 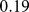 at 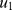, for 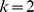, 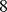 and 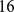, respectively). In lower intensity regions, in particular at 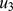, 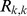 values tended to narrow toward the medial value of the distribution. In the case of shifted distributions (Fig. 3E), true intensities were equal at 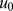 and differed at 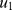, 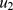 and 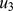, with 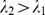. Accordingly, empirical and theoretical curves coincided only at 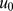 (Kolmogorov-Smirnov test, 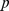-values of 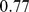, 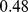, and 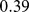 for 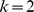, 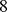 and 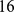, respectively), and we otherwise observed a shift of empirical curves toward smaller values, so that, as expected, the probability to reject 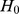 to the benefit of 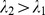 increased (Figs. 3F–H). Note that, as in the homogeneous case, if the CSR hypothesis is acceptable at a position 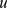, corresponding intensity ratios divided by 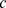, where 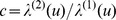, are distributed according to 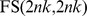. Moreover, 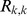 values were once again concentrated when positions moved away from high intensity domains. Finally, although point distributions are heterogeneous, we globally obtained results very similar to the ones in the homogeneous case.

Overall, our method of local intensity comparison is not unduly sensitive to departure from the CSR condition. Consequently, for our local intensity comparison method, the approximation of local point distributions in heterogeneous point processes by a Poisson homogeneous model seems to be an acceptable assumption. Thus, our approach may be reasonably applied to compare intensities in unknown point processes.

#### Systematically mapping intensity comparisons

To illustrate how it can reveal regions with intensity differences in space, our method was applied to compare either homogeneous or heterogeneous point processes. We used 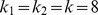 in both cases.

**Figure 4:**
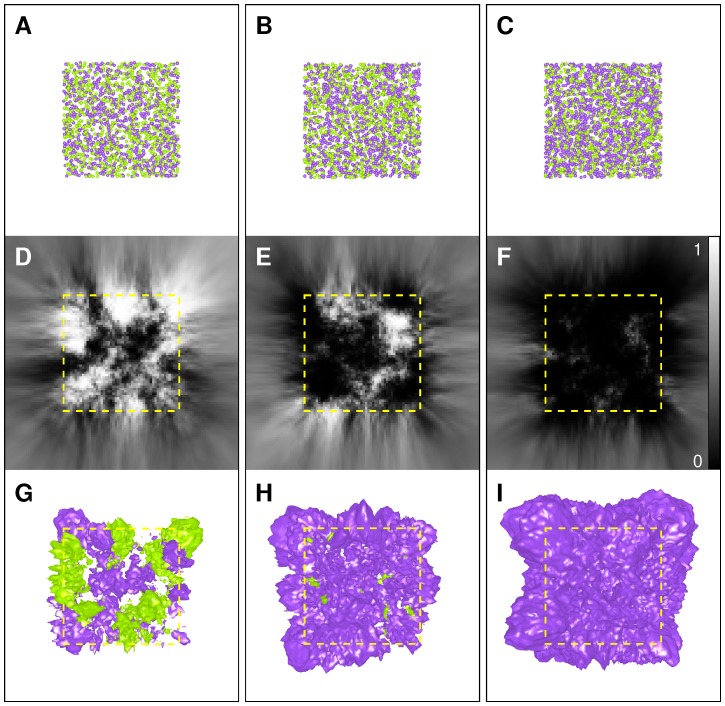
Spatial comparison of homogeneous Poisson point processes. Each column displays data corresponding to a given sample pair (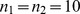). A–C: typical patterns of Sample 1 (green) and Sample 2 (purple), with an average of 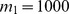 points per pattern, and an average of 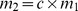 points per pattern, with 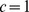 (A), 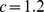 (B), and 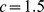 (C). D–F: mid-sections of 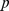-maps computed from Sample 1 and Sample 2, using 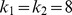. G–I: isosurfaces computed from 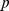-maps for thresholds equal to 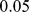 (purple) and 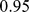 (green) (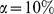). Yellow dotted square in D–I: outline of the unit cube containing generated points.

For homogeneous Poisson processes, comparison maps were calculated over the centered cubic domain of side length 2, using a regular cubic grid of resolution 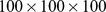. Typical results are shown in Fig. 4. When 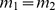 (Fig. 4A), the null hypothesis 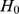 was true at any position. Accordingly, within the central cubic region containing generated points (central region in Fig. 4D), no marked organization emerged in the 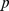-map. Given a risk 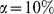, values of less than 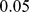 (related to 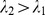) and greater than 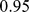 (related to 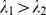) concerned only 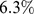 and 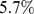 of the 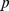-map positions, respectively. These numbers are consistent with expected proportions of 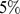. Outside the central unit cube, where the variability of 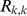 decreased and the probability of not rejecting the null hypothesis increased (see previous Section), the 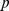-map accordingly exhibited a smoother aspect and was dominated by mid-gray values (see lateral domains in the grid in Fig. 4D). To visualize these results in 3D, isosurfaces were computed from the 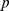-map for 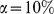 (purple and green surfaces in Fig. 4G, respectively). Unorganized patterns in the 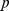-map 2D section appeared as highly intermingled and scattered patterns in 3D, mainly located within the central unit cube. As 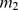 increased (Figs. 4B and 4C), the number of low 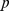-values associated with the hypothesis 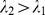 grew accordingly: 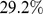 and 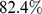 of grid positions within the central unit cube showed a 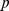-value of less than 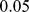 for 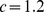 and 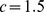, respectively. Conversely, only 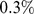 and 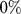 of these positions showed a 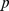-value greater than 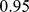, respectively. Such values were expected since, as mentioned above, values of 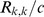 are distributed according to 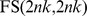. Thus, under the CSR assumption at position 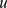 and a given 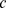, proportions of ratio values less than 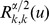 and greater than 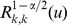 can be evaluated and are equal to 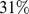 and 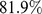 for 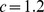 and 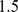, and to 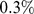 and 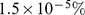 for 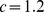 and 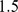, respectively. Finally, corresponding isosurfaces well illustrated the dominance of low 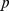-values in the central domain (Figs. 4HI).

**Figure 5:**
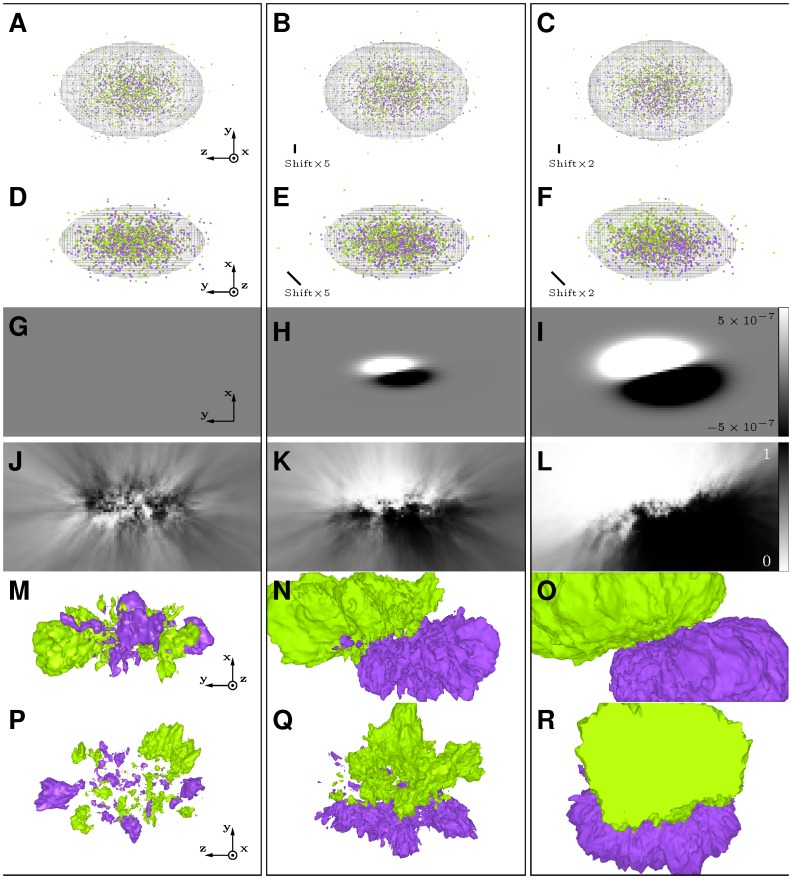
Spatial comparison of heterogeneous point processes. Each column displays data corresponding to a given sample pair (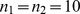). Spatial dimensions corresponding to 3D representations and maps are 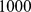, 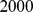 and 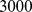 units on the 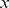, 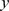 and 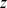 axes, respectively. A–F: typical patterns of Sample 1 (green) and of Sample 2 (purple), with an average of 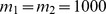 points per pattern. Point coordinates are drawn from Gaussian distributions (same standard deviations as in Fig. 3). Gaussian distribution centers of the two processes are either identical (AD) or shifted by a vector equal to 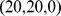 (BE) or to 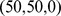 (CF). Meshes: limits of the regions of interest. Patterns are observed either along the 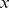 axis (A–C) or the 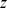 axis (D–F). G–I: mid-section (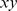 plane) in maps of true intensity differences between first and second point processes. J–L: same section as in G–I in 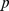-maps computed from Sample 1 and Sample 2, using 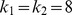. M–R: isosurfaces computed from the 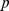-maps for thresholds equal to 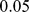 (purple) and 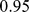 (green) (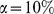). M–O: same viewpoint as in (D–F). P–R: same viewpoint as in (A–C).

For heterogeneous Poisson processes, comparison maps were calculated over a centered rectangular domain using a regular grid of dimensions 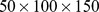 along the 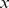, 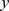 and 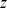 axes, respectively. Typical results are shown in Fig. 5. Since the true point distribution has an unbounded support, we used spatial regions of interest (ROI) to concentrate analyses on high intensity regions. To do this, the domain including higher intensities and containing a given percentage of the underlying distribution function was determined for each process to be compared. The ROI was then defined as the union of the two domains. In this case, ROIs corresponding to 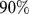 of the spatial point distributions were used (see typical patterns and ROI envelopes in Figs. 5A–F). For identical Gaussian processes (no shift between point distribution centers, first column in Fig. 4), results were analogous to the ones obtained in the case of identical homogeneous processes. First, within the ROI, the 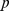-map exhibited unorganized patterns (central ovoid region in Fig. 5J). Second, for a risk 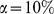, percentages of ROI positions presenting 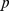-values of less than to 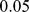 and greater than 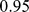 were accordingly equal to 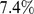 and 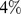, respectively. In contrast, outside the ROI, the map showed a smoother aspect and was dominated by medial values. This reflected the lower variability of 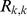 and the increased probability to not reject the null hypothesis in regions of low intensity. In accordance with these observations, isosurfaces extracted from the 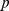-map exhibited rather scattered patterns mainly concentrated in the central region. As for shifted processes (Figs. 5BE and 5CF), differences in spatial organizations clearly appeared in both comparison maps (Figs. 5K and 5L) and superimposed isosurfaces (Figs. 5NQ and 5OR). However, first and second gaps were small when compared to the size of the underlying Gaussian distributions (in the X direction: 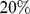 and 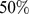, and in the Y direction: 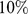 and 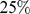 of the standard deviations of the distribution, for the first and second gaps, respectively). Separations between dark and light values in comparison maps related to 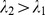 and 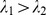 hypotheses, respectively, fitted well with the limit that appeared in true difference maps (Figs. 5H and 5I). For a shift of 20 units in both the X and Y axes and at risk 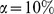, 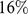 and 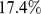 of ROI positions presented a 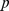-value of less than 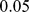 and greater than 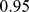, respectively. The percentages reached 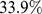 and 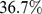 for a shift of 50 units in both the X and Y axes, clearly confirming the gap between processes. The integration of local expected proportions for 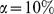 computed at all ROI positions based on true intensity ratios yielded global theoretical proportions of 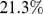 and 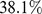 for the first and second shifts, respectively, for both lower (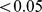) and higher (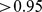) values (proportions are identical due to the symmetry of comparison maps). Empirical values were slightly less than the theoretical ones. This was mainly due to the bias effect observed in low intensity regions, since ROIs encompassed large regions that were poor in data near their borders. Finally, combined with the clear bipolar aspect of 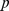-maps, these measures supported the fact that the compared processes were shifted along the direction orthogonal to the transition limit between low and high 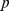-values. Isosurfaces clearly revealed how each process was positioned relative to the other in 3D.

### Application to Neuroanatomical Data

The comparison mapping was applied on two neuroanatomical systems to illustrate the method. First, in the rat, the functional organization of a cerebral structure, the sacral parasympathetic nucleus, was analyzed by describing the relative spatial localizations of two neuronal populations. Next, in the mouse, the effect induced by a mutation over the spatial organization of a neuronal population, the locus coeruleus, was analyzed by comparing data either from control or mutant individuals.

#### Spatial organization of the sacral parasympathetic nucleus

**Figure 6:**
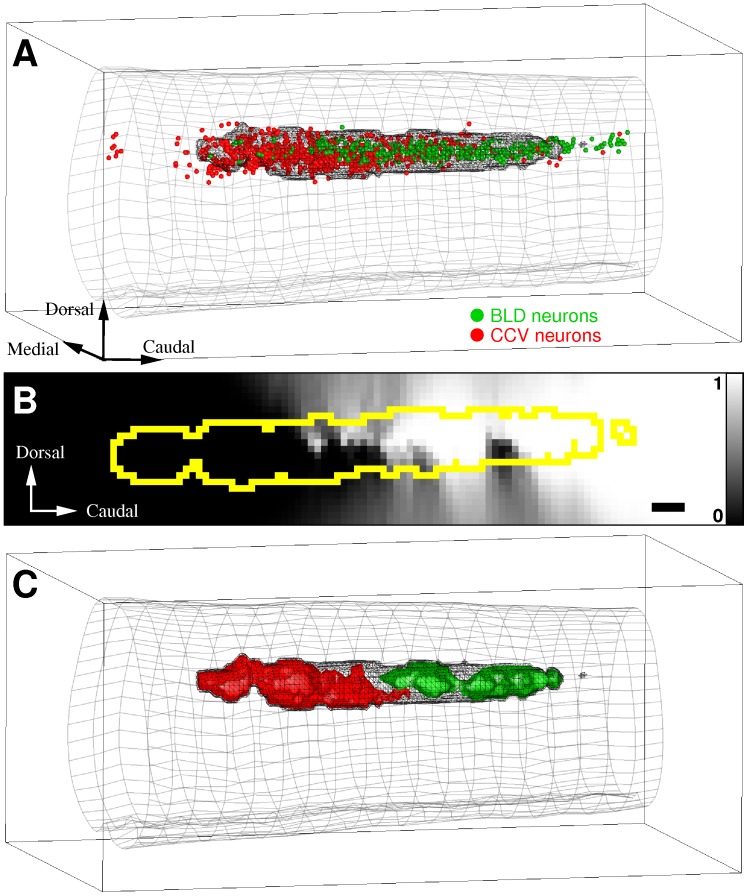
Comparison of functional populations in the rat sacral parasympathetic nucleus (SPN). A: positions of neurons in the SPN innervating either the bladder (BLD, green dots) or the corpus cavernosum (CCV, red dots). Large gray mesh: lumbo-sacral spinal cord envelope. Black mesh: region of interest (ROI) encompassing a majority of neurons. B: mid-section in the 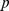-map (lateral plane), with ROI outline in yellow. Scale bar: 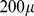
m. C: surfaces encompassing regions where cell intensities are significantly different between the two sub-populations (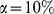, same viewpoint as in A). Surfaces are colored according to the intensity-dominant population.

In the rat, the uro-genital reflex motor activity is controlled by neurons of the sacral parasympathetic nucleus (SPN) within the spinal cord. Banrezes and coworkers previously examined the functional organization of the rat SPN by recording positions of neurons innervating either the bladder (BLD) or the corpus cavernosum of the penis (CCV) [34]. These neurons were retrogradely identified after the injection of a pseudorabies virus in either the penis or the bladder. Spinal cords were then cut into serial sections (30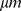 thick) at the level of the SPN and the sections were digitized under light microscopy. Labeled neurons were manually segmented and 3D models of neuronal populations were computed using Free-D software [16]. All experimental procedures are detailed in [34]. By superimposing spatially normalized positions of BLD and CCV neurons (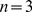 animals per group), a segregation of the two populations along the rostro-caudal axis was observed. The same data were used here to objectively examine previous conclusions and to illustrate our approach on a simple model. Results are presented in Fig. 6. Neuron intensity maps were computed for both groups over a spatial grid superimposed on the data. The grid dimensions were 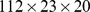 in rostro-caudal, medio-lateral and ventro-dorsal axes, and this corresponded to a grid resolution of 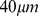. We used the same neighbor rank for both groups, 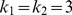. We then restricted our analyses to the region of interest (ROI) of high cell intensities. Since true point distributions were unknown, the ROI corresponded to high intensity domains containing a majority of cells, in this case 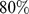 of either BLD or CCV neurons (dark envelope in Figs. 6A and 6C). It corresponded to 2961 of the grid positions (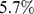 of the total). A medial section through the comparison map is shown in Fig. 6B (the ROI limit is indicated by a yellow contour). Within the ROI, 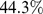 and 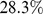 of the positions presented a 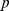-value of less than to 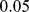 (predominance of CCV neurons) or greater than 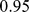 (predominance of BLD neurons), respectively (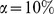). This quantitatively demonstrated the very strong population segregation reported previously [34]. This further revealed that the transition along the rostro-caudal axis between regions dominated by either one or the other population was rather abrupt, as shown by the rapid change between high and low gray values in the 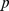-map (Fig. 6B), and by the proximity of opposite comparison isosurfaces that clearly divided the ROI into two distinct regions (Fig. 6C). It is important to note that this rapid transition was unnoticeable when visualizing the simple superimposition of neurons.

#### Locus coeruleus

**Figure 7:**
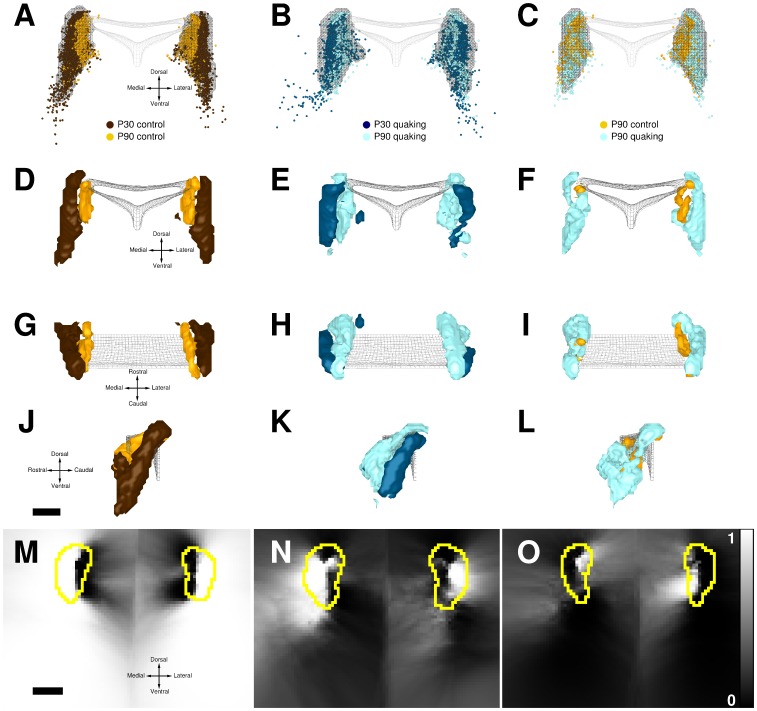
Comparison of locus coeruleus (LC) distributions in control and mutant mice. LC populations at post-natal day 30 (P30) and 90 (P90) for control mice (first column) and quaking mice (second column), and at P90 for control and quaking mice (third column). A–L: the central gray mesh represents the contour of a portion of the fourth ventricle. A–C: superimposed neuron positions (three mice in each group) for control mice at P30 (brown dots) and P90 (orange dots), and for quaking mice at P30 (dark blue dots) and P90 (light blue dots); black meshes: contours of the regions of interest (ROIs). D–L: comparison surfaces including positions where cell intensities were statistically greater in one group (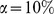), shown from the caudal (D–F), dorsal (G–I) and lateral (J–L) points of view; colors indicate the intensity-dominant group within the surface (same color code as for cells). M–O: coronal mid-sections through 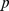-maps, with ROI outlines in yellow. MN: lighter and darker gray levels correspond to positions with either an excess in P30 or in P90 cells, in control (M) or in mutant (N) mice, respectively. O: lighter or darker gray levels correspond to positions with either an excess in control or quaking cells, respectively. Scale bars: 400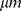.

The locus coeruleus (LC) is a cerebral structure located on both sides of the fourth ventricle. In the mouse, the quaking mutation is known to affect the post-natal maturation of the LC. In particular, between postnatal day 30 (P30) and 90 (P90), the significant decrease of the LC neuron numbers in control mice is not observed in mutants [35]. In this previous study, mouse brains were cut into coronal serial sections (10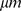 thick) at the level of the LC, and sections were processed for tyrosine hydroxylase immunohistochemistry to identify LC neurons. Sections were digitized under light microscopy, and labeled neurons were manually indicated on images using Free-D software. These same data were used in [29] to analyze the effect of the mutation on the spatial organization of the LC. To do this, 3D models of LC populations were built and spatially normalized, and neuron intensity maps were computed using the intensity estimator 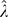, in either control or quaking mice, at P30 and P90 (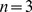 in each group). Then, based on these maps, 3D distributions of LC neurons were visually compared to identify spatial differences during the LC maturation in control and quaking mice. In the present study, comparison maps were built using these intensity maps and were examined in light of previous findings. Results are presented in Fig. 7. ROIs were defined as high intensity domains including 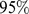 of neurons (meshes encompassing neurons in Fig. 7A–C and yellow contours in Fig. 7M–O). Isosurfaces were generated from 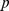-maps using thresholds equal to 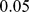 and 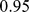 (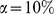).

The maturation process was first assessed by comparing LC distributions between P30 and P90, in both control and mutant mice (first and second column in Fig. 7, respectively).

In control mice, a remarkable right/left symmetry appeared within the 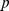-map (Fig. 7M) and in 3D representations (Fig. 7DG). Regions showing a predominance of P30 cells (light pixels in the 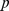-map and surfaces colored in brown) corresponded to ventral and lateral parts of the LC, and involved 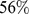 of ROI positions. This is consistent with previous results showing that the decrease in cell number between P30 and P90 mostly concerns these regions. Moreover, we observed that a medial region of the LC, corresponding to 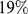 of ROI positions, was dominated by P90 neurons (dark pixels in the 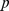-map and surfaces colored in orange). This could not be established beforehand because of the proximity of medial limits in superimposed LC distributions. Thus, based on quantitative arguments, our results show that the LC reorganization between P30 and P90 is not limited to a cell regression in the ventro-lateral parts, but involves a cell increase in the medial regions as well.

In quaking mice, regions corresponding to a dominance in either P30 or P90 cells contained 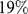 and 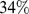 of ROI positions, respectively. These proportions were very different and reversed compared to those obtained for control mice. The comparison map was dominated by dark pixels (Fig. 7N) that reflected the excess of LC cells in P90 mutants. It has been previously shown that the LC maturation between P30 and P90 involves a slight lateral cell regression together with a shift toward the rostral direction, and no change in the dorso-ventral direction [29]. Accordingly, we first observed a quantitative dominance of P30 cells in the lateral parts (light pixels in the 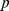-map and surfaces colored in dark blue). Next, P30 cells were more numerous than P90 ones in the caudal region, while the reverse was true in the rostral part (Fig. 7K). Finally, no obvious difference was found in the dorso-ventral direction (Figs. 7E and 7K). As in control mice, we also found a significant dominance of P90 cells in mutants in the medial region (dark pixels in the 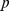-map and surfaces colored in light blue). Altogether, these results show that the decrease in the number of LC neurons in the lateral parts is counterbalanced by an increase not only in the rostral, but also in the medial regions.

The comparison of control and mutant mice at P90 made it possible to reveal in adult mice spatial differences that result from the two distinct LC maturation processes (third column in Fig. 7). It was previously shown that at P90, LC cells in quaking mice extend almost everywhere beyond LC cells in control ones, more especially in the rostral and ventral regions [29]. This conclusion was quantitatively corroborated in our study. First, regions corresponding to an excess of LC cells in mutant animals (dark pixels in the 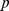-map, surfaces colored in light blue) corresponded to 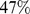 of ROI positions, and generally concerned the ventral and rostral regions (Figs. 7FIL). An excess of LC cells in mutants was also observed in the lateral regions (see lateral parts dominated by dark pixels in Fig. 7O). This lateral excess could not be detected in the previous study. Conversely, only 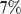 of ROI positions showed a predominance of LC cells in control mice (light pixels in the 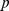-map, surfaces colored in orange), mainly in the medial region. Several observations suggested that this corresponded to non significant effects. In fact, in this region, map and isosurfaces displayed unorganized patterns that were reminiscent of those observed with simulated data in the absence of intensity difference (Figs. 4DG and 5JMP). We also observed a lack of right/left symmetry (Figs. 7FI). Eventually, the small number of corresponding ROI positions was consistent with the threshold of 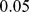. In conclusion, in P90 mice, our results suggested that LC cells extend further in the ventral, lateral and rostral directions in mutants, whereas the medial region is little affected.

## Discussion

We introduced a new approach for the comparison of the spatial organization of two point processes, based on replicated data. Using our method, spatial comparison maps are built that contain 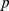-values of local intensity comparisons. These maps can be explored through 2D sections or 3D isosurfaces that encompass positions with significant intensity differences. The main advantage of our strategy is to reveal regions where significant differences occur in terms of point intensity. By contrast, existing statistical approaches only allow to test whether the underlying point processes are globally the same or not (see, e.g., [4], [31]). The method introduced here is an important breakthrough since when comparing experimental groups (e.g., control vs. mutant, young vs. old, or healthy vs. pathological), the localization of 3D regions that show differences is crucial for the understanding of the biological mechanisms affected. Moreover, the fact that comparison results can be analyzed through simple, meaningful spatial representations facilitates biological interpretations. Indeed, the spatial analysis and the objective comparison of punctual structures in 3D is a difficult question. The application of the mapping procedure on simulated data showed that our approach can detect small differences in point distributions (see heterogeneous process comparisons), thus confirming that our method should be a sensitive and powerful tool to unravel complex architectures.

The rank 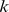 of the nearest neighbor is the only parameter for the intensity comparison. In most cases, the same 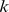 value could be used for both samples to be compared. In the case of intensity estimation, 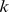 acts as a smoothing parameter [19] and, consequently, has the same effect in the comparison procedure. The choice of 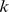 should then depend on the scale at which differences are considered to be relevant. It is interesting to note that we have experimentally shown that the distribution of the ratio 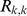 compared to the expected distribution under the null hypothesis remains globally unchanged as 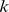 varies. This indicates that results are not too sensitive to the choice of 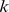. It has been previously experimentally shown that in point intensity mapping, a value of 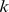 that minimizes the root mean square estimation error exists [19]. This optimal value increases as the true intensity grows, so that 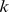 should be chosen according to the mean number of points in pattern samples. High 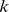 values should be used with precaution: when enlarging the neighborhood taken into account for intensity estimations, the local CSR assumption may no longer be acceptable, especially in low intensity regions. However, simulations done on heterogeneous point processes showed that our method is robust to departure from this working assumption, thus opening the way for its use in biological applications. For a given intensity and a given grid resolution, intensity estimates at adjacent grid nodes are all the more likely to rely on same points that 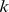 is large, thus implying local correlations between tests. For a given 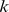 value, correlations are much lower in areas of high intensity, since in this case distances to 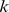
th nearest neighbors are reduced. Altogether, using reasonably low values for 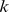 and restricting analyses to the regions containing the majority of the points helps to prevent or minimize correlations.

Using our method for point process comparison, many hypothesis tests are conducted in parallel to build comparison maps. Thus, multiple comparison correction could be considered using, e.g., Bonferroni-like procedures [36] or the false discovery rate controlling procedure [37]. In the present approach, the examination of the proportion of extreme 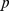-values resulting from comparison tests is crucial to determine if actual differences exist between two point processes. Indeed, using simulated data, we showed that this proportion is consistent with the risk 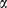 when there is no intensity difference, and that it dramatically increases otherwise. Moreover, simulations illustrated that the structure and the localization of patterns emerging in comparison maps are very informative. We believe that interpreting comparison maps in their entirety for interpretation in biological applications effectively makes up for the absence of correction for multiple testing.

Our method was illustrated on two neurobiological systems. This made it possible to demonstrate the value of the comparison of spatial distributions of punctual data in neuroanatomy. The application of the comparison method to the study of the functional organization within the sacral parasympathetic nucleus provided a precise vision of the relative positioning of two functional populations. Although the spatial organization of this cerebral structure is rather simple, our method revealed in the comparison map a narrow transition area that was undetectable on raw data, since populations were highly intermingled in this region. The spatial architecture of the locus coeruleus (LC) constituted a more complex model to be investigated. The LC is heterogeneous with respect to its projection sites [38]–[40], and its maturation selectively affects specific sub-regions [41], [42]. The effect of the quaking mutation on the spatial LC maturation was analyzed using our comparison method. Our results corroborated those previously obtained by visually comparing neuron distributions based on intensity maps [29]. Moreover, we also pointed out differences that were impossible to establish by comparing point distributions without considering intensities: in both lineages, we showed that the number of neurons between P30 and P90 increases in the medial region of the LC. This illustrated that, by comparing point intensities instead of point distributions, subtle differences can be detected and supported by statistical arguments. We also showed that, in adult mice, an excess of cells in mutants occurs everywhere in the LC except in the medial LC domain. This suggests that this region may not be affected by the quaking mutation. These observations are of importance in the study of biological mechanisms that are impacted by the quaking mutation. In particular, the convulsive phenotype induced by the quaking mutation is directly associated with the excess of LC neurons in the mutant, as demonstrated by the anticonvulsant effect of electrolytic coagulation of the LC [43]. Since specific regions affected by the mutation were revealed in our study, the analysis of their different efferent targets should help to reveal brain regions involved in the convulsive phenotype.

More generally, since cerebral structures are organized into functional areas, the constitution of atlases is a major consideration in human [44] and animal [45], [46] brain research. However, since limits of punctual structures are not easy to define, placing neuronal populations in such atlases is not an easy task. Thus, combined with statistical functional data obtained in neuroimaging, the possibility to determine anatomical regions dominated by specific cell populations defined by functional, physiological or morphological properties, for example, could constitute a major contribution to the development of statistical neuroanatomical atlases.

Finally, our comparison strategy is generic and may be applied to various biological systems and at different scales. It offers statistical and comprehensive spatial representations of differences in point patterns. Consequently, we think that our method meets the need for more quantitative tools devoted to the analysis of spatial organizations, and that it constitutes a novel, efficient and practical tool for the study of biological systems.
